# Severe burns complicated by EDKA, CDI, and NTIS: A case report and literature review

**DOI:** 10.1097/MD.0000000000047272

**Published:** 2026-01-16

**Authors:** Tiantian Yan, Dongsheng Hu, Nannan Wang, Zhichen Lin, Song Li, Miejie Wang, Jin Wen, Ran Liu, Qi Feng, Jing Jia, Ruoyu Shang, Guoan Lin, Rong Xiao

**Affiliations:** a Military Burn Center, The 990^th^ Hospital of the Joint Logistic Support Force, Zhu Madian, Henan, China; b Military Service Office, The 988^th^ Hospital of the Joint Logistic Support Force, Zheng Zhou, Henan, China; c Pathology Department, The 990^th^ Hospital of the Joint Logistic Support Force, Zhu Madian, Henan, China; d Ultrasound Diagnostic Department, 82^nd^ Army Hospital, Baoding, Hebei, China; e Department of Rehabilitation Medicine, The 980^th^ Hospital of the Joint Logistics Support Force, Shijiazhuang, Hebei, China; f State Key Laboratory of Trauma and Chemical Poisoning, Institute of Burn Research, First Affiliated Hospital of Army Medical University (Third Military Medical University), Chongqing, China.

**Keywords:** central diabetes insipidus, euglycemic diabetic ketoacidosis, nonthyroidal illness syndrome, severe burn, type 2 diabetes mellitus

## Abstract

**Rationale:**

Severe burns trigger sustained hypermetabolism marked by protein hypercatabolism, insulin resistance, and relative insulin deficiency. These effects are exacerbated in diabetic patients, increasing susceptibility to acute metabolic crises.

**Patient concerns:**

We report the first case of a burn patient with type 2 diabetes mellitus (T2DM) concurrently presenting with euglycemic diabetic ketoacidosis (EDKA), central diabetes insipidus (CDI), and non-thyroidal illness syndrome (NTIS). Following severe burn and inhalation injury, he exhibited agitation, delirium, and hypotonic polyuria.

**Diagnoses:**

Lab tests confirmed EDKA and NTIS. The EDKA was resolved with standard treatment, but persistent polyuria prompted further evaluation. A positive water deprivation test with desmopressin challenge confirmed CDI, despite unremarkable pituitary MRI.

**Interventions:**

Oral desmopressin acetate and hydrochlorothiazide reduced daily urine output to 3000 mL. Thyroid function normalized post-stabilization. The wound healing was achieved through 2 skin grafts.

**Outcomes:**

The patient received long-term oral administration of metformin, desmopressin acetate, and hydrochlorothiazide. Blood glucose and urine output were well controlled, with no adverse events observed. Only mild scarring was observed, and the patient successfully reintegrated into society and daily life following satisfactory functional recovery.

**Lessons:**

Severe burns disrupt the neuro-immuno-endocrine axis through mechanisms such as stress, inflammation, infection, and inadequate caloric intake. This case highlights the necessity of metabolic monitoring and vigilance for metabolic crises in severely burned patients with diabetes. Key lessons include: EDKA, a rare endocrine emergency, should be suspected in cases of metabolic acidosis/elevated anion gap even in the absence of marked hyperglycemia; Persistent hypotonic polyuria unrelated to blood glucose warrants evaluation for diabetes insipidus, with a water deprivation test with desmopressin challenge distinguishing CDI from nephrogenic diabetes insipidus and osmotic diuresis; NTIS requires treatment only for the underlying condition without specific hormone interventions, though structural or functional thyroid pathologies must be excluded before attributing thyroid dysfunction solely to NTIS.

## 1. Introduction

Euglycemic diabetic ketoacidosis (EDKA), a rare but life-threatening diabetic emergency (incidence 0.8–1.1%), mimics DKA clinically with normal/mildly elevated glucose (<13.9 mmol/L)^[1,2]^ and ketoacidosis (pH<7.3, HCO3^-^<10 mmol/L, ketonemia/ketonuria), often leading to diagnosis delays due to atypical hyperglycemia.^[1,3,4]^ This diagnostic conundrum and delayed treatment may result in serious consequences such as death.

Central diabetes insipidus (CDI) is a syndrome caused by impaired vasopressinergic neurons and defected synthesis and/or secretion of arginine vasopressin peptide (AVP). The AVP deficiency results in impaired reabsorption of water and urine concentration, causing hypotonic polyuria, polydipsia, and low-specific gravity or hypotonic urine.^[5,6]^ The incidence of CDI is approximately 0.004%.^[7]^ Etiologies include hypothalamic/posterior pituitary damage caused by congenital abnormalities, tumors, trauma, autoimmune diseases, inflammation, etc. Additionally, up to 30% of CDI cases are idiopathic without clear cause.^[8]^

Nonthyroidal illness syndrome (NTIS), also known as euthyroid sick syndrome, refers to transient thyroid dysfunction without intrinsic thyroid disease. It is typically triggered by severe non-thyroid systemic illness, surgery, or stress, with abnormal thyroid tests correlating with disease severity and prognosis.^[9,10]^ This article reports the first case of a severely burned patient with type 2 diabetes mellitus (T2DM) who concurrently suffered from EDKA, CDI, and NTIS, highlighting their pathophysiological interplay and management challenges.

## 2. Case report

**Figure 1.**
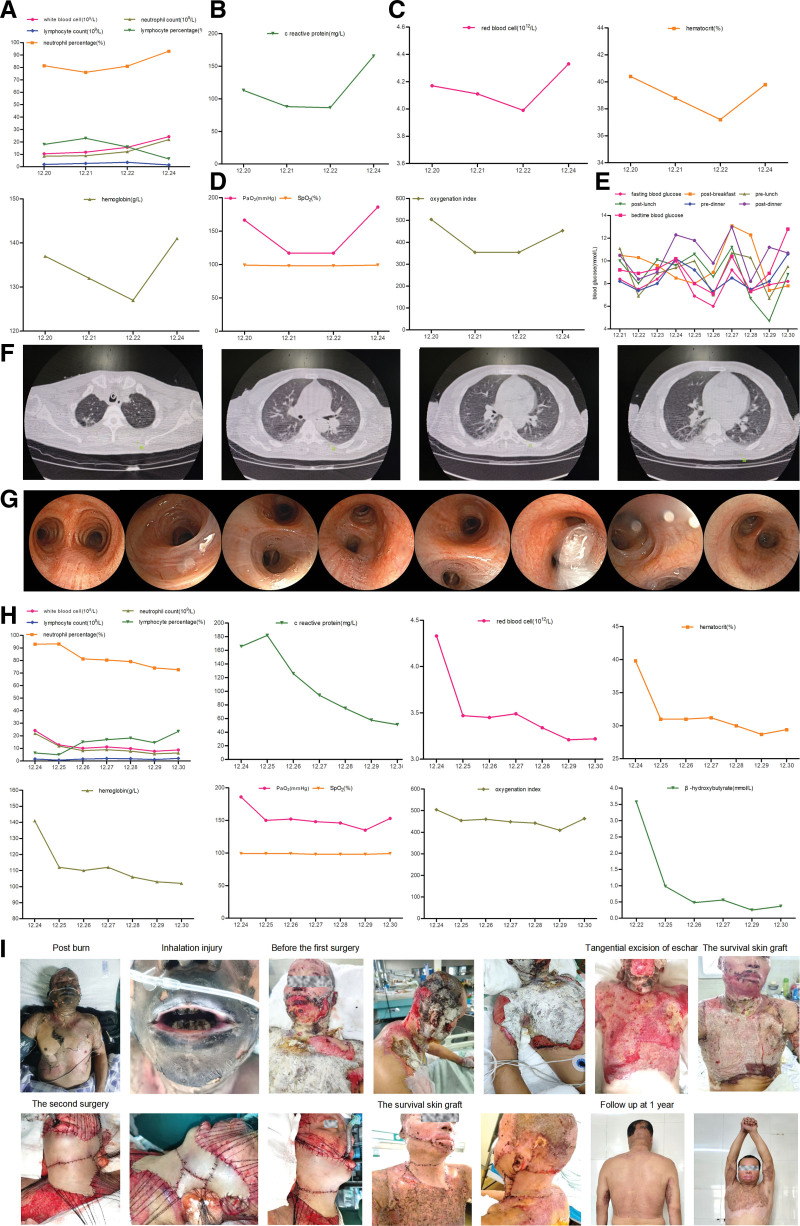
Laboratory results, imaging examination, surgeries, and follow-up of the patient. (A) Complete blood count indicated infection. (B) C reactive protein significantly increased. (C) Complete blood count indicated dehydration and blood concentration. (D) Arterial blood gas analysis indicated good oxygenation. (E) Blood glucose indicated mild elevation. (F) Chest CT confirmed pulmonary infection. (G) Fiberoptic bronchoscopy confirmed mild congestion and edema of the bronchial mucosa with white mucoid secretions. (H) Laboratory results showed improvement after treatment. (I) Two skin graft surgeries achieved full wound healing with mild scarring and no functional impairments.

A 50-year-old male patient was admitted after sustaining flame burns from a liquefied gas leak 6 days ago. The patient remained in the enclosed room for approximately 30 seconds. Initial management included fluid resuscitation and tracheotomy. Current pulmonary examination reveals scattered moist rales in bilateral lower lungs, accompanied by intermittent cough productive of yellow sputum. Inhalation injury was diagnosed based on the injury history, with 21% total body surface area burns (20% full-thickness, 1% deep-thickness deep burns) on his head, face, neck, trunk, and bilateral upper limbs. Although the patient denied any significant medical history, including diabetes mellitus, laboratory findings upon admission revealed a markedly elevated glycated hemoglobin (HbA1c) level of 8.4% (68.3 mmol/mol), consistent with previously undiagnosed diabetes. Additional results included a random blood glucose level of 11.9 mmol/L, elevated C-reactive protein (CRP) (113.05 mg/L) and procalcitonin (PCT) (4.4 ng/mL), while bacterial cultures of blood and wound secretions were negative. After admission, the patient consumed 4000 to 5000 mL of fluids daily, with a hypotonic urine output of 5500 to 7000 mL. On day 3 (post-burn day 9), he developed agitation, acute delirium, consciousness disturbance, tachypnea (26–28/min), and tachycardia (105–120/min). Notable laboratory tests findings included severe metabolic acidosis (pH 7.08, HCO₃^-^3.6 mmol/L, anion gap 33.4, standard base excess -24.1 mmol/L, bicarbonate 3.6 mmol/L, blood lactate 2.3 mmol/L), elevated white blood cell (23.52 × 10⁹/L), neutrophil percentage (93.04%), neutrophil count (21.88 × 10⁹/L), and CRP (165.6 mg/ L) (Fig. 1A, B). Red blood cell count, hematocrit, and hemoglobin levels all significantly increased compared with previous results due to dehydration and hemoconcentration (Fig. 1C). The patient showed normal oxygenation (PaO₂ ≥110 mm Hg, oxygenation index ≥ 350, SpO₂ 98–100%) (Fig. 1D) and slightly elevated blood glucose (7.5–10.3 mmol/L) (Fig. 1E). Bilateral lower lung revealed moist rales, CT confirmed pulmonary infection (Fig. 1F), and fiberoptic bronchoscopy confirmed mild congestion and edema of the bronchial mucosa with white mucoid secretions (Fig. 1G). Cultures of bronchoalveolar lavage fluid identified vancomycin-sensitive multidrug-resistant Staphylococcus lugdunensis. Bacterial cultures from urine, blood, wound, and central venous catheters samples were negative.

Interventions such as intravenous crystalloids resuscitation, sodium bicarbonate, and norvancomycin hydrochloride for suspected sepsis and acidosis yielded limited improvement.

Persistent tachycardia, deteriorated tachypnea (35–40/min), tachycardia (120–135/min), and altered mental status prompted further evaluation. Meanwhile, new laboratory findings revealed urine glucose (+++, 14 mmol/L), urine ketones (+++), and elevated serum β-hydroxybutyrate (β-HB, 3.57 mmol/L). The presence of severe metabolic acidosis (as evidenced by arterial blood gas results), ketosis (characterized by significantly elevated blood β-HB, glucosuria, and ketonuria), and the absence of hyperglycemia collectively fulfilled the diagnostic criteria for EDKA.^[1,2]^ Immediate treatment was initiated, including fluid resuscitation, 5% glucose infusion supplemented with potassium chloride and insulin, and continuous intravenous insulin administration via syringe pump. Blood glucose levels were monitored hourly and maintained at approximately 6 mmol/L. Within 12 hours, the intervention resolved metabolic acidosis, normalized vital signs, arterial blood gas and complete blood count, reduced inflammation, and restored consciousness (Fig. 1H).

**Figure 2.**
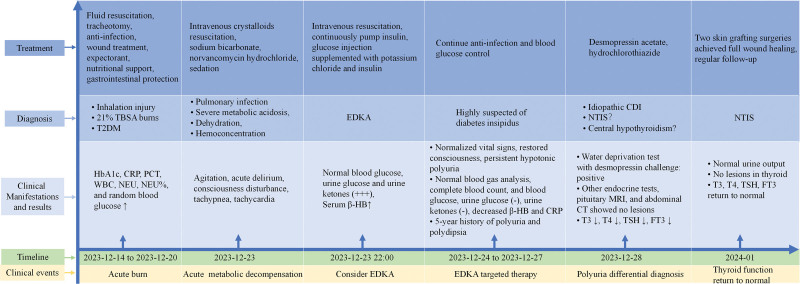
Timeline diagram of disease progression, diagnosis, and treatments. TBSA = total body surface area, T2DM = type 2 diabetes mellitus, HbA1c = glycated hemoglobin, CRP = C-reactive protein, PCT = procalcitonin, WBC = white blood cell, NEU = neutrophil, EDKA = euglycemic diabetic ketoacidosis, β-HB = β-hydroxybutyrate, CDI = central diabetes insipidus, NTIS = nonthyroidal illness syndrome, T3 = triiodothyronine, T4 = total thyroxine, TSH = thyroid-stimulating hormone, FT3 = free triiodothyronine.

Post-EDKA management, the patient exhibited persistent hypotonic polyuria (>5500 mL/day) unrelated to glycemia. A 5-year history of unreported polyuria (5 L/day) and polyuria was collected. The positive water deprivation test with desmopressin challenge confirmed CDI, as evidenced by the elevated plasma osmolality, unchanged urine output/osmolality post-deprivation, increased urine osmolality, decreased plasma osmolality and urine output after pituitrin injection (Table 1). Other endocrine tests, hearing test, pituitary MRI, audiometry, fundoscopy, and abdominal CT were unremarkable, leading to the diagnosis of idiopathic CDI. Oral desmopressin acetate (0.1 mg tid) and hydrochlorothiazide (25 mg qd) decreased urine output to ~3000 mL/day. The patient's thyroid function normalized post-stabilization. Two skin graft surgeries achieved full wound healing with mild scarring and no functional impairments, enabling return to daily activities (Fig. 1I). The patient received long-term oral metformin, desmopressin acetate, and hydrochlorothiazide and no adverse events were found (Fig. 2). This case underscores the diagnostic challenge of EDKA in normoglycemic patients and the importance of evaluating CDI in diabetic burn patients with refractory polyuria.

**Table 1:** Urine output and laboratory findings before and after the water deprivation test with desmopressin challenge.

Time	Date	Hourly urine output (mL/h)	Daily urine output (mL)	Sodium (mmol/L)	Blood pressure (mm Hg)	Plasma osmolality (mOsm/kg)	Urine osmolality (mOsm/kg)
Pre-water deprivation	12.22	235	5520	136	130/84	293.34	250
Post-water deprivation	12.24	216	5185	152.6	120/66	327.16	300
Post-desmopressin injection	12.25	80	1920	137	136/72	297.48	420

## 3. Discussion

Diabetes mellitus (DM), with rising global prevalence, severely impacts life quality. Severe burns exacerbate metabolic dysregulation in DM patients through stress-induced hypercatecholaminemia, glucocorticoid surges, and sustained hypermetabolism, compounded by post-burn insulin receptor dysfunction and impaired glucose uptake, leading to insulin resistance.^[11]^

**Figure 3.**
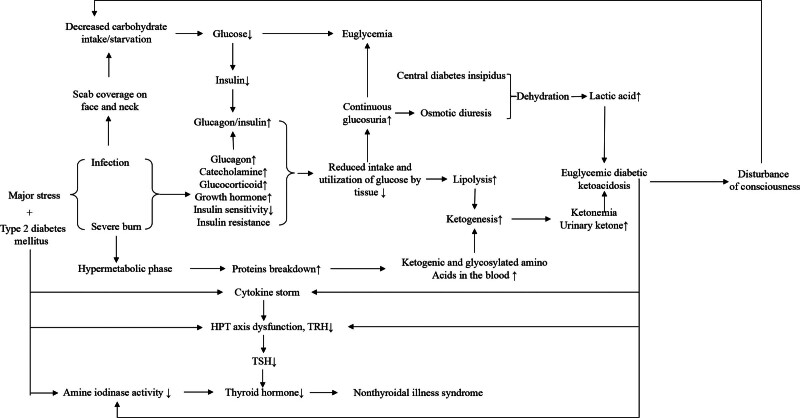
Schematic diagram of the pathophysiological mechanisms underlying the development of the patient's condition.

In this case, facial/cervical eschar restricted oral intake, precipitating carbohydrate deficiency. Post-burn hypermetabolism and infection drove protein catabolism, insulin resistance, and tissue glucose underutilization. Osmotic diuresis (glucosuria) and underlying CDI caused dehydration and glucose loss, triggering compensatory lipolysis (elevated β-HB) and proteolysis (increased gluconeogenic/ketogenic amino acids), culminating in EDKA. The subsequent consciousness disorder further reduced intake and exacerbated energy deficits and dehydration. Initially, EDKA was not identified timely, resulting in delayed diagnosis, worse condition, and coma. Targeted intervention broke this cycle and stabilized the patient (Fig. 3).

Diabetics with preexisting metabolic abnormalities face heightened risks of acute metabolic crises like EDKA and DKA post-burn. Unlike DKA, EDKA lacks characteristic hyperglycemia, increasing diagnostic and treatment challenges.^[2]^ EDKA can occur in patients with T1DM, T2DM, and gestational diabetes, with precipitating factors including reduced caloric intake, alcoholism, infection, chronic liver disease, surgery, pregnancy, and use of sodium-glucose cotransporter-2 inhibitors.^[12–16]^ Future efforts can prioritize the development of EDKA-specific guidelines,^[12]^ clinician education for early recognition, and further investigation of biomarker research (e.g., β-HB elevation as a diagnostic clue to distinguish EDKA from other conditions with similar presentations).^[17]^

This case represents the first reported co-occurrence of CDI, EDKA and severe burns. Key diagnosis and treatment challenges arose from overlapping symptoms and pathophysiological interactions of CDI and T2DM. Symptom overlap: Polyuria and polydipsia, shared by both CDI and DM, masked diagnoses. This patient with normal thirst regulation experienced absence of hypernatremia/hyperosmolality and diagnostic delays.

Pathophysiological interactions: CDI-induced dehydration exacerbates hyperosmolarity, accelerating diabetic ketoacidosis progression. Conversely, diabetic hyperglycemia complicates CDI assessment via osmotic diuresis, obscuring urine output evaluation. In the differential diagnosis of polyuria, although osmotic diuresis shares clinical manifestations with CDI, there are notable differences in laboratory findings due to different pathophysiological mechanisms.^[18]^ The differentiation in this case was based on the following considerations. First, the patient consistently presented with hypotonic polyuria, characterized by low urine specific gravity and urine osmolality. In contrast, osmotic diuresis, due to the presence of substantial glucose in the urine, typically exhibits high urine osmolality and urine specific gravity. Second, diabetic osmotic diuresis is usually accompanied by hyperglycemia and positive urine glucose. In this case, however, blood glucose remained around 6 mmol/L via continuous insulin infusion, without hyperglycemia or glycosuria, thereby ruling out osmotic diuresis. Finally, the water deprivation test followed by vasopressin administration provided critical evidence for confirming CDI. Due to insufficient arginine vasopressin synthesis or/and secretion, patients with CDI fail to concentrate urine after water deprivation, showing high plasma osmolality, low urine osmolality, and persistent polyuria. A positive response, marked by significantly increased urine osmolality, decreased plasma osmolality and reduced urine output following vasopressin administration, supports CDI.^[18]^ In contrast, cases of osmotic diuresis show no such response to exogenous vasopressin.

Etiological links: CDI and T2DM may share pathophysiological pathways, with diabetic vasculopathy potentially damaging the hypothalamic-pituitary portal system, leading to neuronal degeneration in osmoreceptors, supraoptic nucleus, and paraventricular nucleus.^[19]^ Literature review identifies 15 reported CDI-T2DM cases (Table 2), mostly idiopathic and preceding long-standing T2DM,^[8]^ though simultaneous onset is also possible.^[20–32]^ A key common feature between these 13 reported CDI-T2DM cases and our case is the co-occurrence of T2DM and CDI. The primary distinction lies in the sequence of onset: in previously reported cases, T2DM developed and was diagnosed first, followed by a diagnosis of CDI several years later. Although no definitive causal relationship suggesting that “T2DM leads to CDI” has been established, it is hypothesized that CDI may arise from long-term diabetic microvascular complications affecting pituitary perfusion, thereby impairing vasopressin synthesis and/or secretion. Further studies are needed to explore this possibility. In the present case, the patient had a history of polydipsia and polyuria for over 5 years prior to admission, which had not been previously evaluated or intervened. Therefore, it remains unclear whether these symptoms were solely attributable to T2DM or were the manifestation of T2DM and CDI. The temporal relationship and any potential causal relationship between T2DM and CDI in this patient also remain unclear. With advancements in imaging and laboratory technologies, CDI previously diagnosed as idiopathic has now been linked to pituitary or autoimmune or vascular pathologies. Therefore, long-term follow-up and regular MRI examinations are recommended to identify any occult causes that were not detected at the time of diagnosis.

**Table 2:** Cases diagnosed as idiopathic CDI with Type 2 diabetes mellitus in published literature.

Reference	Sex	Age	T2DM before CDI (yr)	Treatment	Associated diseases
Palumbo, 2018^[19]^	F	72	8	Desmopressin Acetate	Dislipemia, Hypertension, prostate cancer
Concepción-Zavalet, 2021^[20]^	M	44	13 yr	chlorpropamide, carbamazepine, hydrochlorothiazide	Vitiligo
Ohara, 2002^[21]^	F	75	5	Desmopressin	Hashimoto thyroiditis
Gossain, 1975^[22]^	M	45	15	Chlorpropamide	Depression, gout
Gossain, 1975^[22]^	M	44	8	Chlorpropamide	None
Paulose, 2002^[23]^	F	37	Unknown	Unknown	None
Akarsu, 2006^[24]^	F	46	10	Desmopressin	None
Gotoh, 2002^[25]^	M	46	Unknown	Desmopressin	Klinefelter syndrome
Isobe, 1992^[26]^	M	41	Unknown	Trichlor methiazide/ Desmopressin	Klinefelter syndrome
Shin, 2012^[27]^	M	56	Unknown	Desmopressin	None
Lowrey, 1950^[28]^	F	7	Unknown	β-hypophamine tannate	None
Engstrand, 1950^[29]^	F	46	Unknown	Death	Acromegaly, hypertension
Sakaguchi, 1998^[30]^	F	37	Unknown	Unknown	Albright hereditary osteodystrophy -like syndrome
Liu, 2005^[31]^	F	22	>4 mo	Desmopressin	None
Cherchir, 2024^[32]^	F	53	Unknown	Desmopressin	Herpes meningoencephalitis
Our case	M	50	Unknown	Desmopressin,	None

CDI = central diabetes insipidus, T2DM = type 2 type 2 diabetes mellitus.

Additionally, this patient also presented with NTIS, evidenced by reduced total triiodothyronine (T3), free T3 (FT3), total thyroxine (T4), and thyroid-stimulating hormone, indicating a critical condition.^[33–35]^ NTIS itself lacks specific clinical manifestations and imaging changes. Thyroid ultrasonography, thyroid uptake rates, and other radiological studies are not diagnostic, and laboratory tests are primarily relied upon for diagnosis. This patient simultaneously presented with severe burns, infections, dehydration, EDKA, and CDI, resulting in critical stress. NTIS is likely multifactorial, including critical illness-induced HPT axis dysfunction, tissue 5’-deiodinase impairment, and aggregated thyroglobulin, altered thyroid follicular structure, infiltrated monocytes, and depleted thyroid follicles.^[36]^ Currently, there remains controversy regarding the supplementation of thyroid hormone in NTIS patients.^[37]^ In our case, despite severe metabolic stressors (burns, infection, EDKA, CDI), thyroid function normalized without hormone replacement. Further large-scale randomized clinical trials are needed to investigate this issue.^[37]^

## 4. Conclusion

We report the first case of a severely burned patient with T2DM concurrently presenting with EDKA, CDI, and NTIS. Severe burns disrupt the neuro-immuno-endocrine axis through mechanisms such as stress, inflammation, infection, and inadequate caloric intake. This case highlights the necessity of metabolic monitoring and vigilance for metabolic crises in severely burned patients with diabetes. EDKA, a rare endocrine emergency, should be suspected in cases of metabolic acidosis/elevated anion gap even in the absence of marked hyperglycemia. Persistent hypotonic polyuria unrelated to blood glucose warrants evaluation for diabetes insipidus, with a water deprivation test with desmopressin challenge distinguishing CDI from nephrogenic diabetes insipidus and osmotic diuresis. Additionally, NTIS requires treatment only for the underlying condition without specific hormone interventions, though structural or functional thyroid pathologies must be excluded before attributing thyroid dysfunction solely to NTIS.

## Author contributions

**Conceptualization:** Tiantian Yan, Dongsheng Hu, Nannan Wang, Zhichen Lin, Song Li, Miejie Wang, Ran Liu, Rong Xiao.

**Data curation:** Tiantian Yan, Dongsheng Hu, Zhichen Lin, Guoan Lin, Rong Xiao.

**Formal analysis:** Tiantian Yan, Dongsheng Hu, Zhichen Lin, Ran Liu.

**Funding acquisition:** Tiantian Yan.

**Investigation:** Tiantian Yan, Dongsheng Hu, Nannan Wang, Miejie Wang.

**Methodology:** Tiantian Yan, Ruoyu Shang, Guoan Lin.

**Project administration:** Tiantian Yan, Qi Feng.

**Resources:** Tiantian Yan.

**Supervision:** Tiantian Yan, Nannan Wang, Jin Wen.

**Validation:** Tiantian Yan, Dongsheng Hu, Nannan Wang, Jin Wen, Rong Xiao.

**Visualization:** Tiantian Yan, Dongsheng Hu, Song Li, Jin Wen, Qi Feng, Ruoyu Shang, Rong Xiao.

**Writing – original draft:** Tiantian Yan, Song Li, Jing Jia, Rong Xiao.

**Writing – review & editing:** Tiantian Yan, Rong Xiao.
